# *Brassica carinata* and *Camelina sativa* oils as renewable raw materials for producing viscoelastic polyurethane foams

**DOI:** 10.1039/d5ra04620c

**Published:** 2025-08-28

**Authors:** Elżbieta Malewska, Danuta Kurasiak-Popowska, Katarzyna Rzyska-Szczupak, Lidia Szwajkowska-Michałek, Krzysztof Polaczek, Federica Recupido, Maria Kurańska, Kinga Stuper-Szablewska

**Affiliations:** a Department of Chemistry and Technology of Polymers, Cracow University of Technology Warszawska 24 31-155 Cracow Poland elzbieta.malewska@pk.edu.pl; b Department of Genetic and Plant Breeding, Faculty of Agriculture, Horticulture and Biotechnology, Poznan University of Life Sciences Wojska Polskiego 28 60-637 Poznań Poland; c Department of Chemistry, Faculty of Forestry and Wood Technology, Poznan University of Life Sciences Wojska Polskiego 28 60-637 Poznań Poland; d National Research Council of Italy, Institute for Polymers, Composites and Biomaterials Piazzale E. Fermi 1, Portici Naples 80055 Italy

## Abstract

This study aims to evaluate the application potential of unrefined vegetable oils derived from three plant species—*Camelina* (*Camelina sativa*), *carinata* (*Brassica carinata*), and rapeseed (*Brassica napus* L. var. *napus*)—as renewable raw materials for the synthesis of bio-based polyurethane foams. The oils, obtained from crops grown in experimental fields in Greater Poland, were first characterized and then chemically modified *via* transesterification with triethanolamine to yield hydroxylated derivatives (biopolyols). As a result of this chemical modification, three biopolyols were obtained, characterized by an average molar mass of ∼500 g mol^−1^, a hydroxyl number of ∼320 mg KOH per g, functionality of ∼2.8 and a viscosity < 200 mPa s. The biopolyols were then used to produce foam materials with viscoelastic properties. The resulting foams had an apparent density of about 70 kg m^−3^, hardness below 2.5 kPa, a support factor (calculated as the ratio of compressive stress at 65% deformation to that at 25% deformation) above 2 and resilience of less than 10%. Additionally, the foaming process of the polyurethane systems containing the newly synthesized biopolyols was analyzed. This study demonstrates the feasibility of utilizing vegetable oils, including non-edible *carinata* oil, as renewable raw materials for the production of sustainable polymeric materials. The results show that, in addition to the widely studied and commonly used rapeseed oil, both *carinata* and *Camelina* oils can also be successfully employed as precursors for the production of bio-based polyurethane foams. Despite differences in fatty acid compositions, the applied synthesis method enabled the preparation of bio-polyols and foams with comparable properties, highlighting the potential of *Camelina* and *carinata* oils as sustainable alternatives to conventional rapeseed oil in industrial applications.

## Abstract 2

Oils from *Camelina sativa*, *Brassica carinata*, and *Brassica napus* as renewable feedstocks for bio-based viscoelastic polyurethane foams.

## Graphical abstract

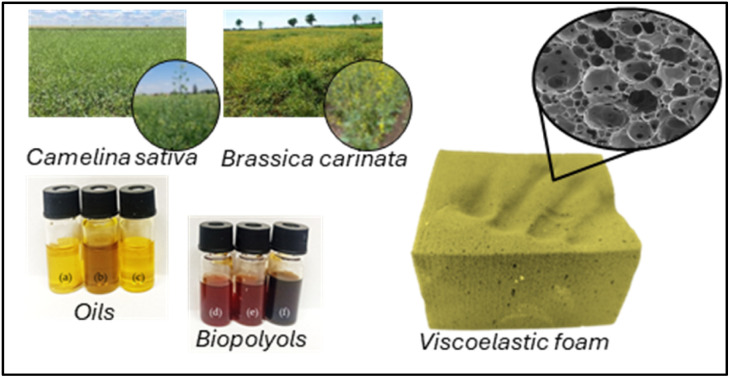

## 1. Introduction

Environmentally friendly technologies are gaining increasing importance in industrial manufacturing, including the polymer sector. Despite this trend, the production of polyurethane materials still relies heavily on crude oil. However, limited fossil fuel resources, fluctuating oil prices, and increasingly stringent environmental regulations compel manufacturers to explore more sustainable and environmentally responsible alternatives. As a result, conventional raw materials and processes are being progressively replaced with renewable resources and innovative, environmentally friendly technologies.^1,2^ In the face of increasing demand for sustainable agriculture, energy independence and reducing greenhouse gas emissions, interest in alternative oil crops is growing. In this context, two plant species, *Camelina* (*Camelina sativa* Crantz) and *carinata* (*Brassica carinata*), are promising species with high potential for industrial use in Poland and Europe.

*Camelina* is cultivated worldwide, primarily for bio-fuel production. In Poland, however, it is traditionally grown for oil production, primarily for food purposes. Winter cultivars grown in Poland are particularly rich in unsaturated fatty acids: α-linolenic acid (ALA, C18:3n-3), linoleic (LA, C18:2n-6), oleic (C18:1n-9), eicosenoic (C20:1). *Camelina* oil finds applications in multiple industries. In the energy sector, it is a raw material for biodiesel and bio-kerosene production because of its high content of polyunsaturated fatty acids and good cold flow properties. In the chemical and food sectors, its balanced lipid composition and high tocopherol (vitamin E) content contribute to oxidative stability and nutritional value. Additionally, its antioxidant and anti-inflammatory components support its use in cosmetics and nutraceuticals. *Camelina* meal, a by-product of oil pressing, can also be used as a protein-rich feed for poultry and fish following appropriate defatting.^3,4^

*Brassica carinata* is a plant species in the Brassicaceae family. Commonly known as “Ethiopian mustard”, “Ethiopian rapeseed”, “Abyssinian mustard”, or simply “*carinata*”, it is developed as a low-emission, non-food oil feedstock for the production of advanced drop-in renewable fuels, protein-rich meals, and bio-based products. This crop presents an interesting non-food alternative to rapeseed, particularly in regions prone to water scarcity.^5,6^

*Brassica carinata* belongs to the oilseed crop group and the oil extracted from its seeds has applications in the fuel industry, most notably as a sustainable aviation fuel (SAF). According to the literature estimates, this fuel can reduce aviation emissions by up to 68% and offers a more cost-effective alternative to traditional kerosene-based aviation fuels. *Brassica carinata* oil contains high levels of undesirable glucosinolates and erucic acid (40–45%), limiting its direct use in food production. Instead, it is utilized in the production of plastics, lubricants, paints, leather tanning agents, soaps and cosmetics.^7,8^

In 2022, *Brassica carinata* was sown for the first time in arable land involved in the implementation of the CARINA project (*carinata* and *Camelina* to boost the sustainable diversification in EU farming systems), conducted under the Horizon Europe program. The project aims to explore the feasibility of cultivating *Camelina sativa* and *Brassica carinata* across Europe, including Poland. *Brassica carinata* and *Camelina sativa* can be grown in simplified systems and regenerative agriculture. Both species can be integrated into low-input cropping systems and are suitable for regenerative agriculture practices because of their pest resistance and soil-improving potential. They contribute to crop biodiversity and exhibit drought tolerance, making them suitable for cultivation in systems focused on maintaining and restoring soil health.

The third oil selected for the study presented in this article is rapeseed (*Brassica napus* L. var. *napus*) oil, playing the role of a reference oil. Rapeseed oil has been extensively described in the literature as a source of natural raw materials for chemical synthesis.^9,10^ Recently, however, its use has been declining because of the increasing emphasis on reducing the use of food resources in chemical synthesis. Consequently, there is a growing need to identify efficient alternatives to rapeseed oil that align with global food policy objectives.

*Brassica napus* L. var. *napus* is widely cultivated in Europe and serves as a source of edible oil as well as a feedstock for biodiesel production and components in chemical synthesis. One of the industries increasingly turning to renewable and waste-derived raw materials is the polyurethane industry.

Polyurethanes are among the most significant polymeric materials owing to their versatility in production and applications. They are primarily manufactured in the form of rigid foams, used in the construction industry for insulation, and flexible foams, commonly applied in the furniture industry.^11^ Other forms of polyurethanes include elastomers, coatings, and adhesives.^12^ Renewable raw materials for polymers are being introduced in response to the challenges of a sustainable economy and environmental concerns.

Viscoelastic polyurethane foams (VFs), a type of flexible polyurethane foam with unique properties, are distinguished by their shape memory effect. These foams are commonly used in the production of high-quality mattresses, pillows, and shock-absorbing materials.^13,14^ Achieving viscoelastic foams with optimal properties requires careful selection of raw materials and precise control of reaction conditions.

The key raw materials for VF production are polyols and isocyanates. Additionally, auxiliary agents such as blowing agents, surfactants, and catalysts are essential components of the process.^15^ Currently, raw materials are predominantly derived from petrochemical sources. However, given the growing concerns over the depletion of carbon and petroleum resources, there is an increasing emphasis on utilizing renewable raw materials, such as vegetable oils and animal fats, as sustainable alternatives.^16^

The substitution of petrochemical polyols with biopolyols is becoming increasingly prevalent.^17^ Various types of vegetable oils, including both edible and non-edible oils, as well as waste oils, can be utilized for the production of polyurethane foams.^18^ Vegetable oils offer several advantages, such as availability, non-toxicity, and renewability.^19^ However, most vegetable oils lack functional groups in their molecular structures capable of forming polyurethane bonds through reactions with isocyanates. To address this limitation, vegetable oils can be chemically modified through several methods, including epoxidation of double bonds combined with oxirane ring-opening reactions,^20,21^ transesterification,^22,23^ alcoholysis^24^ and other methods.^17^

The use of vegetable oils in chemical synthesis, while minimizing their impact on the global food economy, is a critical consideration. Consequently, other environmentally friendly alternatives, such as biomass,^25–27^ waste oils,^28,29^ and recyclates,^30^ have also attracted interest for their application in the production of polyurethanes.

The aim of this study was to evaluate the potential of oils derived from *Camelina sativa*, *Brassica carinata* and *Brassica napus* as renewable raw materials for the synthesis of hydroxyl-functional biopolyols *via* transesterification with triethanolamine, and to assess their applicability in the production of viscoelastic polyurethane foams. The research focused on characterizing the chemical properties of the biopolyols and investigating the influence of their incorporation on the foaming process and the physico-mechanical performance of the resulting foams. To the authors' knowledge, no reports on the production of biopolyols from *Camelina* and *carinata* oils for manufacturing viscoelastic polyurethane biofoams have been published in the literature.

## 2. Materials

### 2.1. Preparation of oils

Three different oils were selected for the study: *Camelina* (*Camelina sativa*) oil (o-CAM), rapeseed (*Brassica napus* L. var. *napus*) oil (o-RAP), and *carinata* (*Brassica carinata*) oil (o-CAR).

The winter cultivar Luna of *Camelina sativa* was registered in the National Plant Breeders' Rights (PBR) in Poland in 2012. The owner of the variety is the Poznan University of Life Sciences. *Carinata* was sown as part of experiments carried out in the CARINA project by farmers in the Greater Poland region on experimental plots. Rapeseed seeds came from experimental crops sown by farmers in Greater Poland.

The experiment was conducted in three replications in three locations in Poland: Kozie Laski (52°21′39′′N 16°11′48′′E); Lubosz (52°30′22′′N 16°09′38′′E) and Przesław (54°18′15′′N 17°04′09′′E) in the growing season of 2023–2024. Each plot had an area of 100 m^2^.

*Camelina* was seeded at a depth of 15 mm using a plot drill. The seed moisture was 9%. The winter *Camelina* cultivar was sown in the third decade of September at a rate of 5 kg ha^−1^. The field management followed standard agricultural practice. Diseases and pests were not combated. The plants were left standing in the field until they were completely ripe. Then, the *Camelina* crops were combine-harvested.

*B. carinata* was sown in March–April, depending on the prevailing weather conditions, with a minimum temperature for emergence of 4–5 °C. The seed moisture was 9%. Well permeable, light-to-medium soils were selected for sowing. The sowing norm was 4–8 kg ha^−1^ and the sowing depth was 1–2 cm. Diseases and pests were not combated. The plants were left standing in the field until they were completely ripe. *B. carinata* was combine-harvested after reaching full maturity.

Rapeseed was sown at the beginning of April. The seed moisture at harvest was 8–10%. The sowing rate was 70–90 seeds per m^2^ (approximately 6–8 kg ha^−1^) and the sowing depth was 1.5–2.0 cm.

**Fig. 1:**
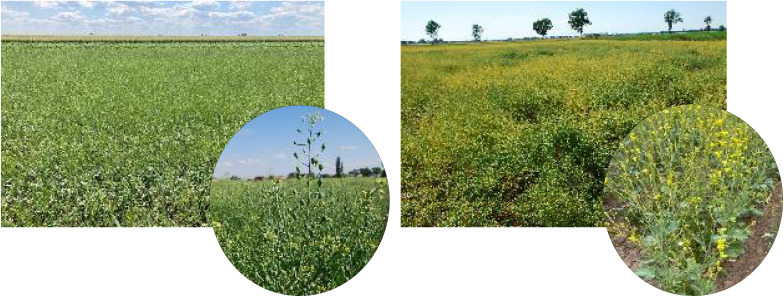
Photographs of the *Brassica carinata* (left) and *Camelina sativa* (right) crops.

Fig. 1 shows the *carinata* and *Camelina* crops on fields.

The implementation of a project under the EUREKA international scientific program ‘*E!4018 CAMELINA-BIOFUEL*’ resulted in the construction of a set of machinery for effective pressing of oil from *Camelina sativa* seeds. The machinery was constructed at the Industrial Institute of Agricultural Engineering in Poznań, Poland. The set consisted of an expeller, a crusher and a screw conveyor, which allowed continuous cold pressing of oil from seeds. After an initial start-up test, which was supposed to warm up the press so that it would operate at a steady temperature, the screw conveyor hopper was filled with a portion of seeds (5 kg) having moisture of about 9%. The seeds were crushed in the 0.2 mm slot of the crusher. During the entire period of the experiment, the average capacity of the crusher was 60 kg h^−1^. The efficiency of the oil pressing process could have been even 69%. Oil pressing from all the seed samples was done at the same time – about one month after the seed harvest. Immediately after oil pressing, seeds and pomace were subjected to physical and chemical analyses.

### 2.2. Materials for biopolyol synthesis

The oils were transesterified using triethanolamine (TEA, pure for analysis) as a transesterification agent and anhydrous zinc acetate (pure for analysis) as a catalyst. Both reagents were provided by Chempur (Piekary Śląskie, Poland).

### 2.3. Materials for the synthesis of viscoelastic foams

Viscoelastic polyurethane foams were synthesized using biopolyols and three different petrochemical polyols supplied by PCC Group (Brzeg Dolny, Poland). The hydroxyl values of polyols 1 (Rokopol® RF551), polyol 2 (Rokopol® M6000), and polyol 3 (Rokopol® M1170) were 410, 29, and 33 mg KOH per g, respectively. The catalyst used in the formulation was Dabco® DC1 (1,4-diazabicyclo[2.2.2]octane supplied by Evonik Industries AG, Essen, Germany). Two surfactants supplied by Evonik Industries were used: Ortegol® 500 as surfactant 1 and Tegostab® B8526 as surfactant 2. Diethylene glycol (DEG) supplied by Chempur was used as a chain extender. Polymeric MDI ONGRONAT® XP 1028 (BorsodChem, Hungary) with an NCO group content of 26.4 wt% was used as isocyanate.

## 3. Methods

### 3.1. Synthesis of biopolyols

Biopolyols were synthesized from the *Camelina*, *carinata* and rapeseed oils through transesterification reactions. In each case, the oil and TEA were introduced into a reactor at a molar ratio of 1:3. The catalyst was added at a concentration of 0.3 wt% relative to the total weight of the oil and TEA. The reaction mixture was heated to 175 °C and stirred at 400 rpm for two hours. The optimal transesterification conditions were achieved based on previous experiments.^31,32^ The products were used as biopolyols without additional processes, such as filtration, purification or by-product separation.

### 3.2. Formulation of viscoelastic foams

The initial reference polyurethane formulation was refined through experimentation. The reference foam formulation is a complex composition consisting of three petrochemical polyols: two with a low OHv of approximately 30 mg KOH per g, and one with a high OHv of 410 mg KOH per g. The polyol with the higher OHv increases the cross-linking density, while the ones with the lower OHv ensure the material's elasticity. In the modified foams, conventional petrochemical polyols were partially replaced with biopolyols at a substitution level of 20 wt%. The level of biopolyol additive was based on previous research, where biopolyols obtained by a similar method were introduced into viscoelastic foams in amounts ranging from 10 to 30%. Using more than 20% of a biopolyol additive was found to impair the properties of viscoelastic foams.^33^ This article focuses on analyzing the impact of the oil type used to obtain biopolyol on the properties of viscoelastic foams rather than on introducing a larger number of variables. To limit the number of variables, only two of the petrochemical polyols were replaced with a new biopolyol: one with a high OHv and one with a low OHv. Specifically, 10 php of polyol 1 and 10 php of polyol 2 were replaced with biopolyol. Based on our experience, the petrochemical polyol 3 helps achieve stable properties in flexible polyurethane foams. Therefore, the amount of this polyol was left unchanged. The isocyanate index was maintained at 0.475 for all formulations. Isocyanate index is the ratio of hydroxyl groups (–OH) to isocyanate groups (–NCO) in a polyurethane system. In the conducted tests, this ratio remained constant, meaning that an excess or deficiency of isocyanate in relation to polyols did not affect the foam properties. To keep the isocyanate index constant, the amount of isocyanate used in each formulation was recalculated, taking into account the change of OHv and the amount of water present in the substrates. The detailed compositions of the individual foams are presented in Table 1.

**Table 1:** Formulations of the viscoelastic polyurethane foams modified with the biopolyols

Component	VF-REF php (per hundred parts of polyol)	VF-CAR php (per hundred parts of polyol)	VF-CAM php (per hundred parts of polyol)	VF-RAP php (per hundred parts of polyol)
Biopolyol	—	20	20	20
Polyol 1	40	30	30	30
Polyol 2	40	30	30	30
Polyol 3	20	20	20	20
Water	3.5	3.5	3.5	3.5
Catalyst	0.6	0.6	0.6	0.6
Surfactant 1	0.5	0.5	0.5	0.5
Surfactant 2	3.0	3.0	3.0	3.0
DEG	2.5	2.5	2.5	2.5
Isocyanate	57.5	60.2	60.0	60.4
Isocyanate index (*I*_NCO_)	0.475	0.475	0.475	0.475

The reference viscoelastic polyurethane foams (VF-REF) and the foams modified with the biopolyols were prepared using a one-step method at 21 °C. First, a polyol premix, containing petrochemical polyols, biopolyols, catalysts, a surfactant and a blowing agent, was thoroughly mixed for 30 s at a speed of 3000 rpm. Then, isocyanate was added and the polyol premix was mixed again for 10 s at speed of 5000 rpm. Finally, the mixture was poured into a plastic mould with a capacity of 1.5 dm^3^.

### 3.3. Oil and biopolyol parameters

The characteristic parameters of the oils were determined in accordance with the following ISO standard methods: acid value (ACv) – ISO 1242:2007, peroxide value (POv) (expressed in milliequivalents of active oxygen per kilogram of oil) – ISO 3960:2007, anisidine value (ANv) – ISO 6885:2006.^34^ The iodine value (IOv) was determined according to the Wijs method following the AOCS Official Method Cd 1-25 (AOCS, 2017). The saponification value (SAv) was measured based on the AOCS Official Method Cd 3-25 (AOCS, 2017). The total oxidation (TOTOX) value was calculated based on the peroxide and *p*-anisidine values following eqn (1).^35^

1 TOTOX = 2POv + ANv

The smoke point of the oils was found in line with the method described by Nielsen.^36^ The biopolyols were also characterized by hydroxyl value (OHv) (in compliance with PN-93/C-89052/03). The density of the oils was measured using a digital densitometer at 20 °C according to the AOAC Official Method 920.212 (AOAC, 2019).

The fatty acid profile (FAME) was also analyzed. Fatty acids were extracted following the method described by Stuper-Szablewska.^34^ Samples containing 100 mg of ground grains were placed into 17 ml culture tubes, suspended in 2 ml of methanol, treated with 0.5 ml of 2 M aqueous sodium hydroxide and sealed tightly. The culture tubes were then placed inside 250 ml plastic bottles and placed inside a microwave oven (Model AVM 401/1WH; Whirlpool, Sweden) operating at 2450 MHz and a maximum output of 900 W. Samples were irradiated (370 W) for 20 seconds and, after approximately 5 minutes, for another 20 seconds. After 15 minutes, the contents of the culture tubes were neutralized with 1 M aqueous hydrochloric acid, then 2 ml of methanol were added and extraction with pentane (3–4 ml) was carried out inside the culture tubes. The combined pentane extracts were evaporated to dryness under a nitrogen stream. In the next step, the extracts were methylated using a mixture of anhydrous methanol and sulfuric acid (1:5, v/v). A lipid-containing extract was added with 0.5 ml of methanol, followed by an addition of a 0.15 ml methanol/sulfuric acid mixture (1:5, v/v). The samples were heated at 70 °C for 15 minutes. After cooling, 0.5 ml of *n*-hexane was added, followed by water in an amount sufficient to form two layers. The upper hexane layer was removed and analyzed using an Aquity H-Class UPLC system equipped with a Waters Acquity PDA detector (Waters, USA). Chromatographic separation was performed on an Acquity UPLC® BEH C_18_ column (150 mm × 2.1 mm, particle size 1.7 μm) (Waters, Ireland). The elution was carried out in a gradient using the following mobile phase composition: A: acetonitrile; B: 2-propanol with a flow rate of 0.17 ml min^−1^. Measurements of sterol concentrations were performed using an external standard at wavelengths *λ* = 195–300 nm. Compounds were identified based on a comparison of the retention times of the examined peaks with that of the standard and by adding a specific amount of the standard to the tested sample and repeating the analyses.

The vegetable oils were subjected to a thermogravimetric analysis (TGA) using a TGA Q550 device from TA Instruments (New Castle, DE, USA). Measurements were conducted in the temperature range of 30–600 °C at a heating rate of 10 °C min^−1^ under a nitrogen atmosphere. The weight of the sample was approximately 10 mg. Based on the TGA and DTG curves, the temperatures corresponding to 5% and 10% mass loss (*T*_5%_ and *T*_10%_), the maximum degradation temperature (*T*_max_), and the residue at 600 °C were found. The determination of molar mass was conducted by gel permeation chromatography (GPC). The GPC analysis of the oils and the biopolyols was conducted using a Knauer AZURA gel chromatograph from KNAUER Wissenschaftliche Geräte GmbH (Berlin, Germany) equipped with a PLgel MIXED-E column for oligomer analysis and a refractometric detector. Calibration was performed following the polystyrene standards. Tetrahydrofuran was used as an eluent at a flow rate of 0.5 ml min^−1^ at 35 °C. The viscosity (*η*) of the oils and the biopolyols was determined at 25 °C using a RM 200 CP40000 PLUS rotational rheometer (Lamy Rheology Instruments).

### 3.4. Properties of the viscoelastic foams

The resulting foams were subjected to a number of tests. As the first step, the foaming process was monitored and analyzed using a FOAMAT® device (Format Messtechnik GmbH, Karlsruhe, Germany). During the foaming process, characteristic times and changes in foam core temperature, pressure and growth rate were recorded as a function of time. The start time was defined as the moment when the derivative of the foam height with respect to time reached 15% of its maximum value. The rise time was defined as the time at which the foam reached 98% of its maximum height. Each foam formulation was analyzed in triplicate.

The morphology of cells was analyzed using a scanning electron microscope TM3000 (Hitachi, Tokyo, Japan) and the software ImageJ (version 1.53f, U. S. National Institutes of Health, Bethesda, MD, USA). Pictures of the foams were taken in cross-sections perpendicular and parallel to the direction of foam growth.

The apparent density (ISO 845:2006), compressive strength (EN ISO 3386-1:1997) and resilience (ISO 8307:2007) were measured in accordance with the relevant standards based on three samples, each measuring 100 × 100 × 50 mm. The samples were analyzed under room conditions at a temperature of 21 °C and air humidity of 60%.

The viscoelastic properties of the foams, including recovery time, were investigated using a RESIMAT® device (Format Messtechnik GmbH, Karlsruhe, Germany). Tests were carried out on three samples taken from each foam formulation at a temperature of 21.5 ± 0.3 °C. The samples with dimensions of 100 × 100 × 50 mm were compressed by 75% of their original height between two pressure plates and maintained in this state for 60 seconds. During compression, the force exerted by the foam on the plates was monitored. After 60 seconds, an immediate clamp release occurred and the return of the foam to its original shape was measured using an ultrasonic sensor, providing continuous tracking of the recovery profile. That procedure allowed determining the ‘appearance’ parameter (unit: mm s), defined in the RESIMAT® method as the area between the recovery curve (sample height *vs.* time) and the original sample height. The integration is performed from the moment of clamp release to the time when the foam reaches 98% of its initial height. This parameter combines both the change in height and the time required for recovery, providing a single numerical value that reflects the recovery profile. Although the unit used (mm s) is not a standard unit in mechanical foam testing, it is applied in this specific measurement method.

## 4. Discussion

### 4.1. Oils and biopolyols

The oils obtained from the seeds of the crops grown in the experimental field were described in terms of their physical and chemical parameters. Table 2 shows the acid profiles of the pressed oils and the content of fatty acid residues of more than 1%. It was found that oils o-CAR and o-RAP exhibit a similar contain of acid residues, *i.e.*, C18:1, C20:1, C22:1 which results in comparable MUFA contents of above 60%. On the other hand, oil o-CAM contains a significantly higher amount of acid residues C18:3n-3 and C20:1, which explains the highest content of PUFA (about 53%). However, in transesterification, the composition of fatty acid residues in the oil is not important, unlike in the production of biopolyols by the epoxidation of double bonds and the opening of oxirane rings. In epoxidation, the number and position of double bonds are crucial.^37^ During transesterification, on the other hand, the presence of double bonds or the chain lengths are less important.^38^ Nevertheless, they may influence the subsequent properties of viscoelastic foams. Most fatty acid residues (92–94%) in the analyzed oils contain between 18 and 22 carbon atoms.

**Table 2:** Acid profiles of the oils

Oil symbol	o-CAR	o-CAM	o-RAP
Acid residue	Acid residue content, %		
C16:0 (palmitic acid)	3.8	4.8	4.4
C16:1 (palmitoleic acid)	—	—	—
C17:0 (heptadecanoic acid)	2.3	—	—
C18:0 (stearic acid)	1.9	3.0	1.6
C18:1 (oleic acid)	28.9	15.9	38.8
C18:2n-6 (linoleic acid)	11.3	11.5	15.5
C18:3n-6 (*y*-linolenic acid)	—	—	7.6
C18:3n-3 (linolenic acid)	14.4	41.9	—
C20:0 (arachidic acid)	1.1	1.0	—
C20:1 (*c*-11-eicosenoic acid)	9.5	18.8	12.2
C20:2 (eicosadienoic acid)	1.1	—	—
C22:1 (erucic acid)	24.1	1.8	18.8
MUFA	62.50	36.5	69.80
PUFA	26.80	53.4	23.10
SFA	9.10	8.8	6.00
Rest	1.6	1.3	1.1

The main characteristics of the oils obtained are summarized in Table 3. The seed oil pressing efficiency was 45% for *Camelina* and rapeseed, and 50% for *carinata*. Unfavorable changes to oils can begin in the oilseed and are unavoidable during oil production. The determined IOv correspond to the presented fat profiles of the oils. The *Camelina* oil has the highest IOv (145 g I_2_/100 g) due to its high PUFA content. The other oils, o-CAR and o-RAP, have a IOv of approximately 120 g I_2_/100 g. Also, the oils have a similar SAv ranging from 189 to 193 mg KOH per g. The resulting oxidation and degradation products of fats can be identified through chemical analysis. The following indices are of particular interest: POv, ACv, ANv and TOTOX. It is generally accepted that the TOTOX value should be below 26. Values below 10 are indicative of superior product quality and freshness.^39^ Products with a higher TOTOX value may be less suitable for regular consumption. All the studied oils met the standards in terms of acid and peroxide values.^40^ For all the oils, ACv is below 5 mg NaOH per g. The POv and ANv indices are slightly higher for o-RAP than those found for o-CAR and o-CAM. However, the TOTOX value is below 10 for all of them. For oils used in chemical processes, these limits are less significant than in the case of edible oils.

**Table 3:** Properties of the oils

Oil properties	Unit	o-CAR	o-CAM	o-RAP
ACv	mg NaOH per g	5.1	3.5	2.9
POv	mg O_2_ per kg	0.48	0.60	1.30
Smoke point	—	234	164	130
ANv	—	0.52	0.63	3.85
TOTOX	—	1.48	1.83	6.45
IOv	g I_2_/100 g	121	145	116
SAv	mg KOH per g	189	191	193
Density	g cm^−3^	0.91	0.92	0.91
Oil pressing efficiency	%	50	45	45

The smoke point is the lowest temperature at which a heated oil begins to break down into glycerol and free fatty acids, losing all its nutritional properties in the process. The smoke point of an oil mainly depends on its free fatty acid content. The more free fatty acids an oil contains, the lower its smoke point. The higher the smoke point of a given oil, the more resistant to decomposition its fatty acids are. Once this threshold is exceeded, the fat starts to smoke and gives off an unpleasant odor.^41^ The oil with the highest smoke point temperature of 234 °C was extracted from *carinata*, whereas the oil with the lowest smoke point temperature of 130 °C was o-RAP. The smoke point temperature of the rapeseed oil corresponds to that reported in the literature for unrefined oils.^40^ However, refined rapeseed oil usually has a smoke point of about 230 °C.^42^

**Fig. 2:**
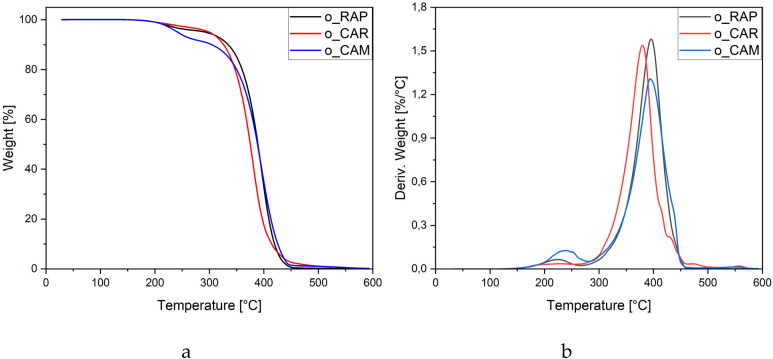
Thermogravimetric (a) and derivative thermogravimetric (b) curves of the unrefined vegetable oils (o-RAP, o-CAR, o-CAM) obtained under nitrogen atmosphere.

The thermal stability of the three unrefined vegetable oils was subjected to a thermogravimetric analysis (TGA) under a nitrogen atmosphere. The thermal degradation of triglycerides in vegetable oils is a complex, multi-step process primarily influenced by the fatty acid composition. Typically, polyunsaturated fatty acids (PUFAs) degrade first, followed by monounsaturated fatty acids (MUFAs), while saturated fatty acids (SFAs) decompose at the highest temperatures. In unrefined oils, natural components such as phospholipids, free fatty acids, tocopherols, and sterols can further influence the degradation behavior.^43,44^ Based on the TGA curves (Fig. 2a), two major stages of weight loss were identified. Key thermal degradation parameters (*T*_5%_, *T*_10%_, *T*_max_ and residue at 600 °C) for each oil are presented in Table 4 to facilitate comparison. The first stage, occurring between approximately 200 and 300 °C, is the most pronounced for o-CAM and less so for o-RAP. It can be attributed to the evaporation of volatile low-molecular-weight fractions, including free fatty acids, or the thermal degradation of natural minor components prior to the onset of triacylglycerol decomposition.^43^ In contrast, highly refined vegetable oils typically show negligible mass loss below ∼300 °C.^45,46^ The second stage, observed between 300 and 470 °C, corresponds to the breakdown of ester bonds in triacylglycerols and the subsequent decomposition of fatty acid chains. The DTG curves (Fig. 2b) confirm a comparable thermal decomposition range for all three oils (300–470 °C), although subtle shifts in the temperature of maximum degradation rate are observed: 379 °C for o-CAR, 395 °C for o-CAM, and 398 °C for o-RAP. These differences are consistent with the fatty acid compositions of the oils (Table 2). The rapeseed oil (o-RAP), exhibiting the highest thermal stability, contains a high proportion of monounsaturated fatty acids (∼69.8%) and a relatively low PUFA content (23.1%). The *Camelina* oil (o-CAM) degrades at a slightly lower temperature, which can be attributed to its very high PUFA content (∼53.4%). Its thermal stability is partly moderated by the MUFA fraction (∼36.5%), but remains lower than that of o-RAP due to a much higher share of thermally labile polyunsaturated fatty acids. In contrast, the *carinata* oil (o-CAR) exhibits the lowest thermal stability, which can be attributed to its high content of long-chain MUFA (∼62.5%), particularly erucic acid (C22:1, ∼24.1%), combined with a considerable proportion of PUFA (∼26.8%). The predominance of very-long-chain unsaturated fatty acids tends to decrease thermal stability, as extended hydrocarbon chain is more susceptible to thermal scission.^47^ The low residue values (0.12–0.25%) indicate almost complete decomposition of organic matter under the TGA conditions and a minimal inorganic content.

**Table 4:** Thermal degradation parameters of the unrefined vegetable oils. *T*_5%_ and *T*_10%_ correspond to the temperatures at which 5% and 10% weight loss occur, respectively. *T*_max_ represents the temperature at the maximum rate of mass loss (DTG peak)

Sample	*T*_5%_ (°C)	*T*_10%_ (°C)	*T*_max_ (°C)	Residue at 600 °C (%)
o-RAP	292	334	398	0.12
o-CAR	299	326	379	0.25
o-CAM	241	304	395	0.23

**Fig. 3:**
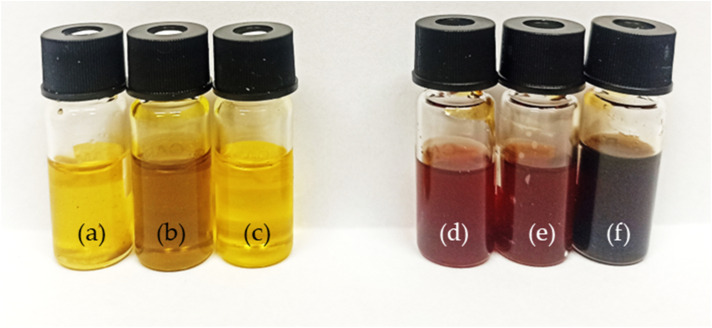
Oil and biopolyol samples; (a) o-CAM; (b) o-CAR; (c) o-RAP; (d) bp-CAM; (e) bp-CAR; (f) bp-RAP.

The pressed oils were chemically modified to obtain hydroxyl derivatives that have the potential to become components in the reaction of polyurethane formation. For this purpose, the oils were subjected to a transesterification reaction using triethanolamine as a transesterification agent. The same reaction conditions were used for each type of oil. As a result of the reaction, biopolyols were obtained and their physico-chemical properties were characterized. The properties of the starting oils and the biopolyols obtained are presented in Table 4. Following the modification, liquid, homogeneous, phase-stable products were obtained, with hydroxyl values between 310 and 350 mg KOH per g and functionalities between 2.7 and 3.0. The oils and the biopolyols obtained are shown in Fig. 3.

The biopolyols obtained had a lower molecular weight (465–505 g mol^−1^), indicating that the transesterification reaction had taken place. The transesterification reaction produces a mixture of compounds. Transesterification is an equilibrium reaction, reversible and takes place in steps.^48^ The reaction mixture consists of mono- and diesters, and glycerol.^49,50^ An appropriate choice of process conditions will shift the equilibrium of the reaction towards monoesters, a high content of which is most desirable.^32^ The OHv, viscosity and molar mass confirm that the biopolyols obtained could be used to produce polyurethane materials.

The efficiency of the transesterification reaction was analysed after two hours using GPC. The percentage contents of individual reaction products (*i.e.* monoesters, diesters, glycerol and substrates that did not undergo the reaction) were determined by this method. The end products of the transesterification reaction are monoesters of fatty acid and glycerol, as well as monoesters of fatty acid and TEA. Table 5 shows that the amount of monoesters in the post-reaction mixture for all biopolyols was approximately 50%. This value is similar to that obtained for monoesters in transesterification reactions carried out under similar conditions using other oils to produce biopolyols.^51,52^ The transesterification reaction efficiency can be increased under different conditions, primarily by using an excess of the transesterification agent, as is done in biodiesel production.^16,53,54^

**Table 5:** Selected properties of the oils and the biopolyols^a^

	OHv, mg KOH per g	*η*, mPa s	*M*_n_, g mol^−1^	*M*_w_, g mol^−1^	*D*	% mE, %	*f*	% H_2_O, %
o-CAR	0	65	960	997	1.03	—	0	0.1
o-CAM	0	50	910	950	1.04	—	0	0.1
o-RAP	0	55	930	960	1.03	—	0	0.1
Bp-CAR	320	180	505	628	1.23	50.2	2.7	0.4
Bp-CAM	310	180	480	589	1.25	50.5	2.8	0.2
Bp-RAP	325	140	465	573	1.24	48.8	2.8	0.3

a OHv – hydroxyl value; *η* – viscosity; *M*_n_ – number average molar mass; *M*_w_ – weight average molar mass; *D* – dispersity of molar masses; % mE – monoester content in biopolyol; *f* – functionality.

As a result of transesterification, the viscosity of the resulting polyols was higher than that of the original oils. The viscosity increased from ∼50–65 to ∼140–180 mPa s. The obtained biopolyols had an OHv ranging from 310 to 325 mg KOH per g. Similar values for molar mass, hydroxyl number and viscosity were obtained in analogous experiments using other oils as the starting materials, *e.g.* fruit seed oil, radish oil and cooking oil.^51,55^ These findings are consistent with previous studies showing that the structure of the starting oil has a limited effect on the main transesterification outcomes when TEA is used as a reagent. This chemical process produces biopolyols, which can be used to synthesize polyurethane materials. To avoid interference with global food production, it is advisable to choose oils from plants that are easy to grow, have a high pressing capacity and are non-edible, or oils from plants that can be grown on degraded or post-industrial land.

### 4.2. Viscoelastic foams

It was found that the hydroxyl oil derivatives could be used in the synthesis of polyurethane materials. A decision was made to develop systems for the synthesis of viscoelastic foams characterized by shape memory. The systems for the synthesis of these materials are described in Table 1. Typically, at least two different polyols with different hydroxyl numbers (high and low) are used in such a synthesis to obtain viscoelastic properties.^33,56^ This type of a polyol system makes it possible to obtain a material in which there is a so-called phase separation responsible for the specific properties of viscoelastic foams.^57^ Three different petrochemical polyols were used in the formulation, as well as a biopolyol added at a level of 20%. The reference foam did not contain any biopolyol. In the first stage, the investigation concerned the effect of the biopolyol addition on the foaming process, which is crucial in foam materials as it affects the quality of the final cell structure.

#### 4.2.1. Foaming process

**Fig. 4:**
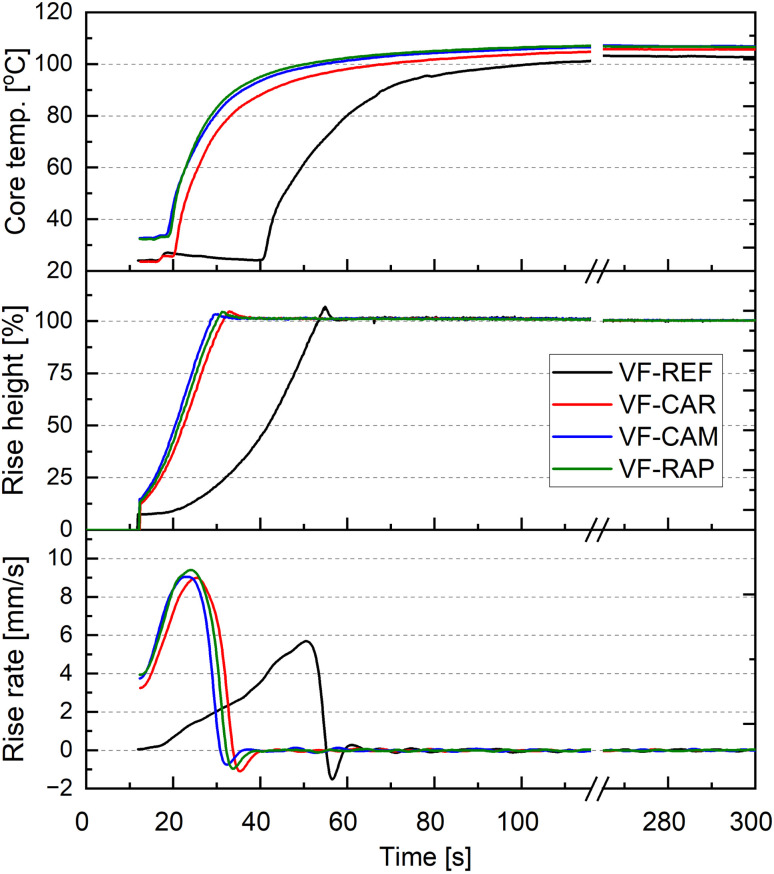
Changes in core temperature, rise height and rise rate during the foaming process, depending on the type of polyol used to produce viscoelastic foams: VF-REF – reference foam, VF-CAR – foam based on bp-CAR, VF-CAM – foam based on bp-CAM, VF-RAP – foam based on bp-RAP.

The foaming process was analyzed using a FOAMAT instrument. The changes in temperature, growth height and growth rate are shown as a function of time (Fig. 4). It was observed that the addition of a biopolyol to the polyurethane system increased the temperature inside the foam core and increased the growth rate of the material. Those changes were observed regardless of the type of the biopolyol used. The presence of triethanolamine (both the free one, which has not reacted, and the one incorporated into the polymer chain) has a catalytic effect on the reactions that take place during the foaming process,^58^*i.e.* the reaction between water and isocyanate. Therefore, when the biopolyols were used, an increase in the growth rate of the foam material was observed compared to the system with petrochemical polyols only.

Similar observations have been described by Kurańska and other researchers. An addition of a biopolyol obtained by triethanolamine-based transesterification increased the reactivity of the polyurethane system.^59^ When using biopolyols obtained from vegetable oils by other methods, *i.e.* epoxidation of double bonds and oxirane ring opening, an inverse relationship was observed.^60^

The characteristic times for the foaming process are shown in Table 6. The systems containing biopolyol had a start time of below 12 s compared to 20 s for the reference foam. The rise time was also about 20 s shorter than in the case of the petrochemical foam. Start and rise times are related to the foaming reaction. Gel time is related to the speed at which isocyanate and polyol react. Also in this case, a reduction of the gel time from 33 s for the reference foams to about 18 s for the biofoams was observed. The maximum temperature in the foam core was around 105 °C and was only slightly higher for the systems containing biopolyols. The foaming analysis showed that replacing 20% of the petrochemical polyol with a biopolyol had a significant effect on the foaming process. This phenomenon can be explained by the presence of TEA molecules incorporated into the biopolyol chain. Various catalysts are used to control the reaction during the production of polyurethane foams. Depending on the catalyst or the system used, the balance can shift from the polyurethane chain formation reaction (gelation) to the water-isocyanate reaction (foaming). Tin salts are typically employed as catalysts for gelation, whereas tertiary amines are utilized for foaming.^61^ Therefore, the presence of many TEA molecules in the chain causes the biopolyol to act autocatalytically, accelerating the foaming reaction and influencing the foam formation process. The use of a biopolyol obtained by transesterification with triethanolamine may also have a positive ecological impact, as the amount of catalysts required in the polyurethane system can be reduced.

**Table 6:** Characteristics of the foaming process of the biopolyol-modified viscoelastic foams

	Start time, s	Rise time, s	Gel time, s	Max. velocity, mm s^−1^	Max. temperature, °C	Shrinkage ratio, %
VF-REF	20 ± 1	60 ± 3	33 ± 2	5.7 ± 0.3	105 ± 1	4.4 ± 0.3
VF-CAR	<12	31 ± 2	18 ± 1	9.4 ± 0.5	106 ± 2	3.8 ± 0.2
VF-CAM	<12	30 ± 1	19 ± 2	9.0 ± 0.2	105 ± 2	3.3 ± 0.3
VF-RAP	<12	29 ± 3	17 ± 2	9.0 ± 0.4	106 ± 1	3.2 ± 0.4

#### 4.2.2. Foam cell structure

**Fig. 5:**
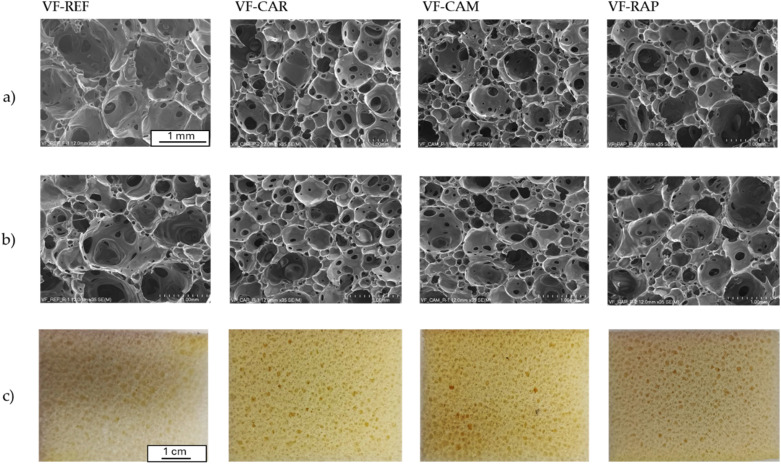
(a) SEM microphotographs taken in a direction perpendicular to foam growth; (b) SEM microphotographs taken in a direction parallel to foam growth; (c) foam photographs.

The foam materials were obtained in accordance with the formulations in Table 1 and then evaluated for their cellular structure. Fig. 5 shows scanning electron microscope (SEM) images of the cellular structures of the foams taken perpendicular and parallel to the direction of foam growth. Additionally, Fig. 5c shows the general appearance of the foams, captured with a standard camera, to demonstrate their uniform cellular structure and defect-free quality. A visual inspection of the SEM micrographs reveals that the obtained foams differ in their cell structure. An image analysis was performed to confirm this observation. Table 6 presents the results of this analysis, *i.e.* the average cell surface area and the number of cells per 1 mm^2^.

The analysis of the cell structures (Table 7) shows that the reference foam has the largest cells. Adding a biopolyol resulted in a finer cell structure. This was particularly noticeable in the foams obtained from BP-CAM, which had cells with an approximate surface area of 0.12 mm^2^. The surface area of the cells in the reference foam was 0.53 mm^2^. Foam VF-CAM had the highest cell density (5.97 cells per mm^2^), while foam VF-REF exhibited the lowest cell density (1.78 cells per mm^2^). The difference can be explained by the presence of a biopolyol that acts as a surfactant. This effect has also been described in previous studies.

**Table 7:** Characteristics of foam cell structures

	Perpendicular direction		Parallel direction	
	Cell area, mm^2^	Number of cells, cell per mm^2^	Cell area, mm^2^	Number of cells, cell per mm^2^
VF-REF	0.53	1.78	0.24	2.83
VF-CAR	0.15	5.13	0.16	5.13
VF-CAM	0.12	5.97	0.12	5.66
VF-RAP	0.22	3.67	0.17	3.98

#### 4.2.3. Physical and mechanical properties of the viscoelastic foams

The next stage of the research was to determine the physical and mechanical properties of the foam materials obtained. The results concerning the compressive strength, resilience and apparent density are summarized in Table 8. It was observed that all the foams obtained had a similar apparent density of between 65 and 70 kg m^−3^, which is typical of viscoelastic polyurethane foams used in industry.^62^ Although a slight increase in density was observed in the foams modified with the biopolyols compared to the petrochemical foam, it was found that those foams were softer. Typically, an increase in the apparent density of a foam material is accompanied by an increase in hardness at 40% foam deformation. In this case, however, the different chemical structure of the polyol components obtained from natural oils resulted in the plastification of the polyurethane matrix. This effect is usually caused by long hydrocarbon chains that are not terminated by functional groups and are therefore known as ‘dangling chains’.^63^ Such groups are present in polyols obtained by transesterification and are incorporated into the polymer structure. The biopolyol-modified foams were characterized by better viscoelastic properties and higher energy absorption capacity. This was evidenced by reduced resilience from 11.6% for VF-REF to ∼9% for the foams derived from the biopolyols, as well as by an increase in hysteresis, which rose from 56.7% for VF-REF to 60.7–64.1% for the biopolyol-modified foams. However, these changes are not significant, meaning the materials can still be classified as viscoelastic.

**Table 8:** Apparent density, hardness, support factor, hysteresis and resilience of the viscoelastic polyurethane foams containing the biopolyols

	Apparent density, kg m^−3^	Hardness, kPa	Support factor	Hysteresis, %	Resilience, %
VF-REF	65.7 ± 1.5	2.5 ± 0.4	2.2 ± 0.1	56.7 ± 2.4	11.6 ± 0.9
VF-CAR	69.3 ± 2.8	2.2 ± 0.2	2.2 ± 0.3	63.0 ± 6.1	9.6 ± 0.9
VF-CAM	70.8 ± 1.3	2.4 ± 0.2	2.3 ± 0.3	60.7 ± 5.7	9.6 ± 0.9
VF-RAP	67.1 ± 0.7	2.2 ± 0.0	2.2 ± 0.4	64.1 ± 4.6	8.4 ± 0.9

Support factor is an index used to determine the quality of foam cushioning, particularly in mattresses and other polyurethane foam materials. It is calculated by determining the ratio of the force needed to deflect the foam by 65% to that needed to deflect it by 25%. A high support factor indicates that the foam has a high load-bearing capacity and is more resistant to deep deflection. This means that, even under pressure, the foam retains its cushioning properties, so the user does not feel as though they are sinking into it. The ideal support factor value is assumed to be 3, but most mattresses on the market have a value ranging from 1.5 to 2.6.^64–66^ The obtained foam materials modified with the biopolyols have a support factor of approximately 2.2. This means that initially, the user experiences softness, but when further loaded, the material provides adequate support to prevent sinking into the mattress.

**Fig. 6:**
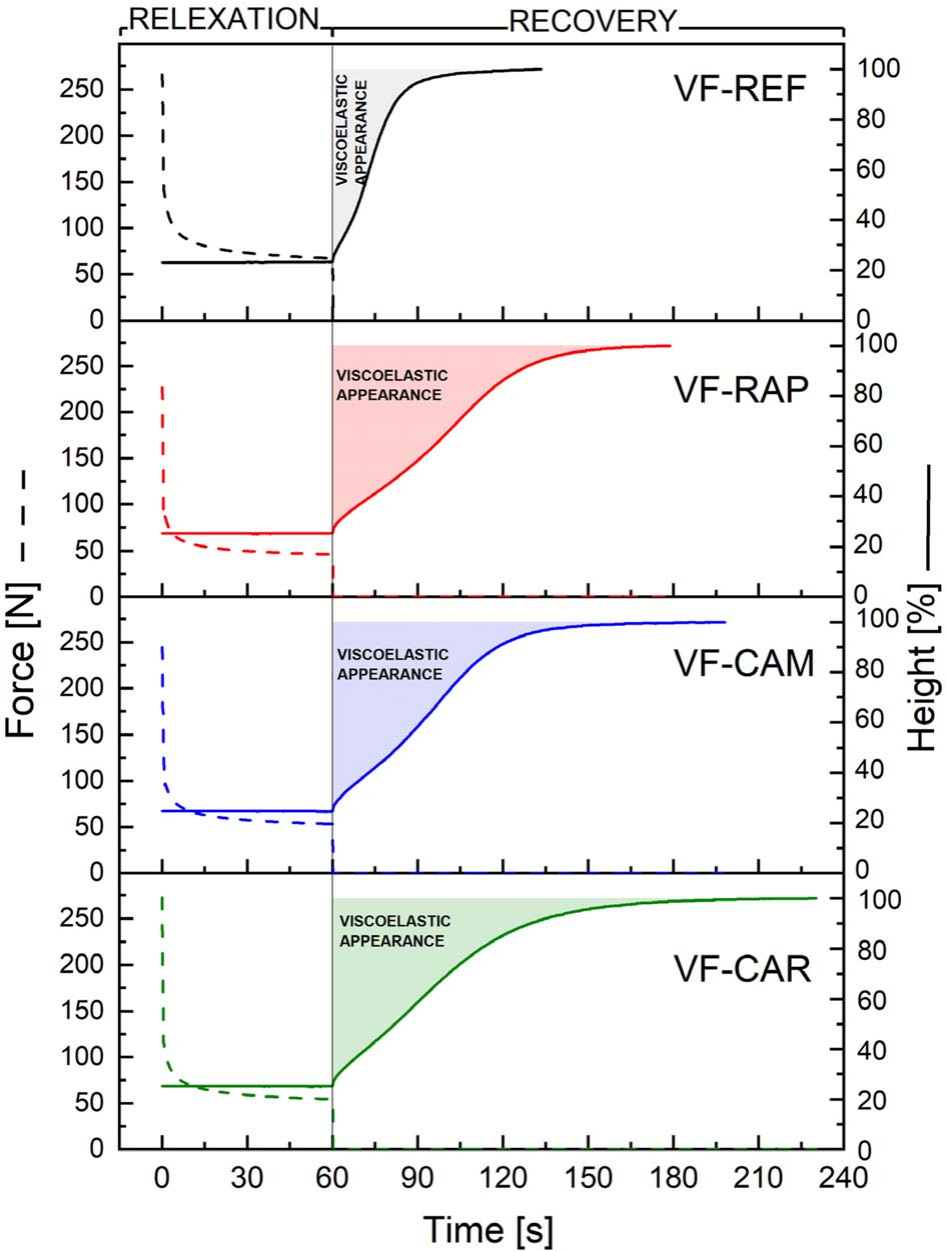
Viscoelastic properties of the foams measured with RESIMAT®.

Viscoelastic foams exhibit characteristic creep behavior when subjected to an external force. This makes them ideal for use in bedding and seating applications. Fig. 6 presents the viscoelastic behavior of the tested foams, while Table 9 lists the numerical results from the RESIMAT® device.

**Table 9:** RESIMAT® results for the foams

	Start force, N	Final force, N	Height 80%, s	Height 95%, s	Max velocity, mm s^−1^	Appearance, mm s
VF-REF	257.7 ± 8.1	64.0 ± 3.1	19.9 ± 0.3	39.4 ± 3.2	3.7 ± 0.1	700.8 ± 42.7
VF-CAR	257.3 ± 4.6	51.7 ± 2.7	52.9 ± 1.5	99.9 ± 0.8	2.7 ± 0.1	1508.7 ± 37.8
VF-CAM	253.8 ± 9.2	52.6 ± 0.6	48.4 ± 0.1	78.3 ± 2.2	3.2 ± 0.2	1391.7 ± 2.8
VF-RAP	225.9 ± 0.8	47.1 ± 0.9	54.1 ± 0.9	85.4 ± 2.2	2.6 ± 0.1	1524.2 ± 44.0

The analysis reveals a clear distinction between the reference sample (VF-REF) and the foams modified with the biopolyols, whereas the differences within the group of the modified foams (VF-CAR, VF-CAM, VF-RAP) remain relatively minor.

The start force values were comparable across all foam samples, with only a slight deviation observed for VF-RAP, which exhibited a noticeably lower initial force. This difference may be partially attributed to its lower apparent density compared to the other bio-based formulations (Table 8). In contrast, the final force measured after a 60 second compression period varied more significantly. The reference foam (VF-REF) showed a force drop of approximately 75.2%, while the modified foams exhibited more pronounced decreases: 79.9% for VF-CAR, 79.3% for VF-CAM, and 79.2% for VF-RAP. These results indicate a greater extent of stress relaxation in the modified formulations, which is consistent with a more viscoelastic mechanical response.

The recovery phase, analyzed based on the time required to reach 80% and 95% of the initial height, further differentiates the behaviors of the materials. The reference foam (VF-REF) exhibited a fast and elastic response, recovering to 80% of its original height in approximately 20 s, while the modified foams required more than twice as long (up to ∼54 s in the case of VF-RAP). Within the group of the bio-based formulations, the differences in the time needed to reach 80% recovery were relatively small, with a spread of only 5.7 s. In contrast, the time to reach 95% of the initial height shows a greater variation among the modified foams, with differences reaching 21.6 s. This suggests that the initial recovery kinetics (up to 80%) distinguishes the reference foam clearly from the modified ones, whereas the final stage of shape recovery (from 80% to 95%) occurs with greater variability even within the bio-based group – particularly between VF-CAR (99.9 s) and VF-CAM (78.3 s).

The maximum recovery velocity supports these trends, with the highest velocity observed for VF-REF (3.7 mm s^−1^), confirming its elastic, rapid-response character. The modified foams demonstrated lower velocities (∼2.6–3.2 mm s^−1^), reflecting a slower and more damped recovery process, characteristic of viscoelastic foams.

The appearance parameter further highlights the differences in viscoelastic response. Viscoelastic foams, which are a subclass of flexible polyurethane foams, are designed to recover slowly, and thus a higher appearance value is desirable to ensure a gradual recovery and memory effect. In contrast, conventional flexible foams, especially high-resilience foams, typically have a very low appearance value, as their recovery is almost immediate after the load is removed, providing high elasticity and rapid shape restoration. Foam VF-REF showed a significantly lower appearance value (∼700 mm s), indicating a short and fast recovery phase. The modified foams exhibited considerably higher appearance values (∼1390–1520 mm s), confirming their suitability for applications targeting memory or damping effects.

The physical and mechanical analyses show that the biopolyols can partially replace petrochemical polyols in the viscoelastic foam production, with only slight changes to the properties compared to the reference foams. The production of viscoelastic foams involves the use of polyols with different hydroxyl numbers. This means that a 20% share of a biopolyol with an LOH above 300 mg KOH per g could be an interesting environmentally friendly alternative to petrochemical polyols.

## 5. Conclusions

Oils obtained from plants cultivated in Greater Poland – *Brassica carinata* and *Camelina sativa* – were identified as promising materials with high potential for industrial use in Europe. Since *Camelina* and *carinata* oils are not intended for consumption, but offer high pressing efficiency, they can be successfully utilized as renewable raw materials in chemical synthesis. Rapeseed oil was used as a reference raw material, and the results indicated that the type of vegetable oil had only a limited influence on biopolyol production *via* transesterification. The monoester yields from the transesterification process catalyzed with triethanolamine were similar for the rapeseed oil and the newly tested *carinata* and *Camelina* oils, at around 50% each. The obtained biopolyols had an OHv ranging from 310 to 325 mg KOH per g and a viscosity in the range of 140–180 mPa s. The hydroxyl number of the biopolyols was within the range commonly found in large-scale commercial products, particularly those used in the production of rigid polyurethane foams. However, the lower viscosity of the biopolyols compared to that of commercial petrochemical polyols can facilitate polyurethane processing. Transesterification process enabled the production of biopolyols that can serve as effective components in polyurethane synthesis. To avoid competition with food resources, it is advisable to select oils derived from high-yielding and non-edible crops that are easy to grow.

The hydroxylated derivatives obtained from the oils were characterized and found to possess OHv and viscosities suitable for polyurethane applications. Physico-mechanical testing confirmed that the biopolyols can partially replace petrochemical polyols in the production of viscoelastic foams, with only minor performance changes compared to the reference materials. A positive effect of using 20% biopolyol, especially from *carinata* and *Camelina* oils, on the cell structure was observed. That was evident from a reduced cell surface area and a higher cell density. The biopolyol presence resulted in a slight decrease in foam hardness (2.2–2.4 kPa) compared to that of the reference foam (2.5 kPa). Also, a hysteresis rise was observed from 56.7% for reference viscoelastic foam to 60.7–64.1% for the biopolyol-modified foams. The changes are related to the chemical structure of the biopolyol with long hydrocarbon chains. Since the synthesis of viscoelastic foams typically involves a combination of polyols with varying hydroxyl values, a 20% replacement with a biopolyol exhibiting an OHv above 300 mg KOH per g appears to be a promising bio-based substitute for conventional polyols with reduced environmental impact, keeping the desired mechanical properties of foams.

## Conflicts of interest

The authors declare no conflict of interest.

## Data Availability

The data presented in this study are available on request from the corresponding author.
